# The concentration-independent effect of arbuscular mycorrhizal fungi on the tolerance of green foxtail to vanadium stress

**DOI:** 10.3389/fpls.2025.1592931

**Published:** 2025-05-27

**Authors:** Shujuan Zhang, Yuexiao Dong, Jingfan Qi, Jinlong Wang, Ze Xi, Ziwei Cao, Kinjal J. Shah, Zhaoyang You

**Affiliations:** ^1^ College of Urban Construction, Nanjing Tech University, Nanjing, China; ^2^ Nanjing Yuqing Environmental Technology Co., LTD., Nanjing, China

**Keywords:** heavy metal, phytoremediation, chlorophyll, ultrastructure, antioxidant system, AMF

## Abstract

**Introduction:**

Arbuscular mycorrhizal fungi (AMF) show significant potential for improving plant tolerance to vanadium (V) stress. However, the pattern and physiological mechanisms behind this effect are not fully understood.

**Methods:**

To investigate this, we used green foxtail (*Setaria viridis*) as a test plant and inoculated this plant with (+AMF) or without (-AMF) *Rhizophagus irregularis*. These +AMF and -AMF plants were grown in soils with low (150 mg kg^-1^), medium (500 mg kg^-1^), and high (1000 mg kg^-1^) V pollution levels.

**Results:**

Our results showed root colonization of +AMF plants, whereas no such colonization was observed in -AMF plants. Compared to -AMF plants, +AMF plants showed a more organized arrangement of leaf cells, intact chloroplasts, fewer starch granules, and an intact nuclear membrane. AMF increased leaf chlorophyll a concentration by 49% under high V pollution and that of chlorophyll b by 18% under low V pollution and 36% at medium soil V levels. AMF reduced the concentration of malondialdehyde (MDA) by 36%-40% in leaves and increased the activities of superoxide dismutase (SOD) by 20%-84%, catalase (CAT) by 5%-13%, and peroxidase (POD) by 12%-16%. +AMF plants exhibited 13%-32% greater plant height, 17%-23% longer root length, 42%-78% higher shoot biomass, 61%-73% greater root biomass, 16% increased root-to-shoot ratio (at high V pollution), and 7%-13% elevated leaf phosphorus concentration than -AMF plants. Furthermore, +AMF shoots had 16%-30% lower V concentrations than -AMF plants while +AMF roots exhibited 52%-73% smaller V concentrations than the -AMF control.

**Discussion:**

These results suggest that AMF increase plant tolerance to V stress by protecting leaf ultrastructure, increasing chlorophyll concentration, reducing oxidative damage as well as biomass-driven V dilution and these effects of AMF were independent of soil V concentrations.

## Introduction

Vanadium (V) is a silver-gray metal commonly used as a raw material in industries (Imtiaz et al., 2015; Li et al., 2023). The discharge of solid, liquid, and gaseous wastes containing V has resulted in V pollution of soil (Teng et al., 2011; Xiao et al., 2015; Yin et al., 2022; Fu et al., 2024). V concentrations greater than 50 mg L^-1^ in the soil solution have been reported to be toxic to watermelon (Nawaz et al., 2018), 30 mg L^-1^ to rice (Yuan et al., 2020), and 10.9 mg L^-1^ to green foxtail (Aihemaiti et al., 2019). Therefore, one of the biggest challenges for plant remediation technologies for V-polluted soils is improving plant tolerance to V stress (Yuan et al., 2024).

Green foxtail has stronger tolerance to multi-metal pollution (V, cadmium, lead, copper, and zinc) and higher biomass than other tested plants such as *Chenopodium album*, *Daucus carota* and *Rorippa indica* (Aihemaiti et al., 2017). The roots of green foxtail do not exist alone; they form symbiotic structures with various microorganisms, such as arbuscular mycorrhizal fungi (AMF) (Zhang et al., 2017). Importantly, AMF have great potential to improve plant tolerance to V and play this role by improving plant nutrition (Zhang et al., 2024). They can form symbiotic structures with their host plants in soils with low (El-Alam et al., 2018), medium (Selim et al., 2021), and high (Qiu et al., 2021) V concentrations. However, it remains unclear whether the effect of AMF on enhancing the tolerance of green foxtail is consistent in soils with low, medium, and high V pollution.

When the stress of heavy metals exceeds a certain threshold, the plant’s cellular ultrastructure is damaged (Esposito et al., 2012). AMF could confer plant tolerance to heavy metals by protecting plant cellular ultrastructural. For example, inoculation with *Rhizophagus intraradices* enables plant (*Potentilla sericea*) roots to maintain more intact cellular structures like cell wall, mitochondrion, and vacuole (Feng et al., 2023). Inoculation with *Rhizophagus irregularis* alleviates cell deformation and collapse of plant (*Eucalyptus grandis*) roots caused by cadmium toxicity (Kuang et al., 2023). In addition to the protection of root cells, inoculation with *Rhizophagus intraradices* (Wang L. et al., 2023) or *Funneliformis mosseae* (Li et al., 2020) protected the ultrastructure of leaf cells. Importantly, no difference in this role between the *Rhizophagus intraradices* and *Funneliformis mosseae* was observed (Li et al., 2020). Consequently, it is hypothesized that AMF can improve V tolerance of the green foxtail by protecting the ultrastructure of plant cells. However, no supporting evidence has been found so far.

Under heavy metal stress, most genes involved in chlorophyll biosynthesis exhibited down regulation and genes related to chlorophyll degradation showed upregulation (Li et al., 2023). Importantly, inoculation with *Glomus mosseae* significantly induced the expression of two enzymes involved in the chlorophyll biosynthetic pathway (coproporphyrinogen oxidase and Mg-protoporphyrin IX chelatase) (Li et al., 2023). A higher concentration of chlorophyll was found in the plants inoculated with *Rhizophagus irregularis* compared to the non-inoculated control under chromium stress (Hu et al., 2020). Inoculation with *Funneliformis mosseae* also increased leaf chlorophyll concentration under lead stress and pre-domesticated AMF strain was more efficient in increasing plant chlorophyll concentration than the non-domesticated control (Yang et al., 2022). However, under V stress, there is no evidence showing that AMF increase leaf chlorophyll levels for green foxtail plants.

Excess V increases the concentration of reactive oxygen species by impairing the activities of antioxidant enzymes and induces the production of malondialdehyde (MDA) which serves as a marker of lipid peroxidation (Kohli et al., 2017; Kumar et al., 2022). It is important to note that under V stress, inoculation with *Funneliformis mosseae* significantly reduced MDA concentration and increased antioxidant activities (SOD, POD and CAT) in leaves (Qiu et al., 2021). Similarly, inoculation with *Rhizophagus irregularis* in C3 (rye) and C4 (sorghum) plants improved redox homeostasis under V stress and higher activities of CAT, SOD and POD) were recorded for rye not for sorghum (Selim et al., 2021). However, it remains unclear whether AMF alter the redox homeostasis of green foxtail under V stress.

As far as we know, it is not clear whether and how AMF improve V tolerance to low, medium, and high V stress. That’s why we set out to answer the following questions:

Question 1: Do AMF enhance plant tolerance to V at low, medium, and high soil V levels?

Question 2: How do AMF induce the tolerance of green foxtail plants to V stress?

## Materials and methods

### Experimental design

The arbuscular mycorrhizal fungus *Rhizophagus irregularis* was used as the test strain and the green foxtail (*Setaria viridis*) was used as the test plant to establish the mycorrhizal symbiosis. The experiment included three V stress levels: low (150 mg V kg^-1^), medium (500 mg V kg^-1^), and high (1000 mg V kg^-1^). At each V level, plants were inoculated with (+AMF) or not (-AMF). Each treatment was repeated five times.

### Seed preparation

Green foxtail seeds were purchased from Beijing Lvzhijie Ecological Technology Development Co., Ltd. related. Healthy, uniform, undamaged and non-mouldy seeds were selected. The seeds were rinsed three times with deionized water and then surface sterilized by soaking in 10% hydrogen peroxide for approximately 10 min, followed by a thorough rinse with deionized water.

### AMF inoculum

AMF inoculum, *Rhizophagus irregularis*, was from the Shanghai Institute of Biological Sciences, Chinese Academy of Sciences. The percentage of root length colonization of AMF was 22%, the density of extraradical mycelium was 0.042 meters per gram, and the spore density was 23 spores per gram.

### Substrate preparation

A mixture of soil and quartz sand in a ratio of 4:6 served as the test substrate. The soil was from the Changshu Agricultural Ecological Experimental Station of the Chinese Academy of Sciences (31°32′55″N, 120°39′24″E). The substrate contained 1.13 g kg^-1^ total nitrogen, 10.6 g kg^-1^ soil organic carbon and 0.35 g kg^-1^ total phosphorous at the beginning of the experiment. The V concentration of the original soil was not detectable. The soil was air dried and passed through a 2 mm sieve. Quartz sand was washed to remove surface dust, packaged in sterilization bags, and sterilized in an autoclave at 121°C for 2 h. The sterilized materials were then cooled, dried, and stored for further use.

The nutrient-rich soil purchased from the Xingyue Flagship Store consisted of imported peat, imported low-salt coconut coir, perlite, nitrogen-phosphorus-potassium fertilizer (N-P_2_O_5_-K_2_O: 15-15-15 and the total nutrient ≥45%), vermiculite and chlorophyll nutrients. We added 0.2 kg of nutrient soil per pot to promote plant growth. In the substrate, 1000 mL of a prepared sodium metavanadate solution (NaVO_3_) was added to adjust three V stress levels: low (150 mg V kg^-1^), medium (500 mg V kg^-1^) and high (1000 mg V kg^-1^). The soil was mixed manually to ensure homogeneity and then left to age for 30 days.

Plastic pots purchased from Zhuyayao Flagship Store were used as containers. The dimensions of the pots were 16 cm in upper diameter, 12 cm in lower diameter and 11.5 cm in height. The pots were sterilized with 75% ethanol and plastic trays were placed at the bottom of each pot. The aged substrate served as a growth substrate, with each pot containing 1.0 kg of the substrate. For the +AMF treatment, 0.3 kg of AMF inoculum was layered into the substrate, while the -AMF control received an equivalent amount of substrate without AMF. Sterilized, pre-germinated green foxtail seeds (only with plumule not the seedlings) were planted with the top layer covered with 0.2 kg of medium. The pots were placed in a random arrangement under natural light with a shelter, changing their position every two weeks and rotating each pot randomly. Irrigation was done every five days, with each pot receiving 100 ml of water per session.

### Plant sampling

After three months of growth, the shoot and root parts of the plants were harvested. The shoot was washed with distilled water to remove adhered soil particles and contaminants. Plant samples were then soaked in a 0.01 M EDTA solution for 30 min to remove heavy metal ions adsorbed on the surface. After blotting dry with absorbent paper, the fresh weight of each plant part was recorded. Fresh samples were used to measure root length, chlorophyll concentration, ultrastructure, MDA concentration, SOD activity, CAT activity, and POD activity. The roots were then excised and fresh root samples were saved for subsequent determination of AMF colonization. The remaining plant samples were first inactivated at 105°C for 30 minutes and then dried in the oven at 70°C until a constant weight was reached. The dried samples were used to measure plant biomass, root-to-shoot ratio, phosphorus concentration, and plant V concentration.

### Sample analysis

Plant height was measured from the soil surface to the tip of the longest leaf using a flexible ruler. The average height for each pot was calculated using the measurements of all plants in that pot. Root length was measured with a flexible ruler from the base of the plant to the tip of the longest root. The average root length for each pot was determined from measurements of all plants in that pot. The fresh weight of each plant part was recorded. The dry weight was then recorded. The root: shoot ratio was calculated by dividing the root dry weight by the shoot dry weight.

AMF colonization of plant roots was determined according to the method (Giovannetti and Mosse, 1980). The root segments were dipped in a 10% potassium hydroxide (KOH) solution and placed in a boiling water bath for 30 minutes to clarify. When the roots became light yellow or transparent, they were rinsed with distilled water to remove any KOH residue. The cleaned root segments were carefully transferred to a 2% hydrochloric acid (HCl) solution and soaked for 5 minutes. Residual HCl was washed off with distilled water. The root segments were then dipped in a 0.01% acid fuchsin-milk glycerol solution and placed in a constant temperature water bath at 90°C for 30 min for staining. AMF were then examined under a microscope at 200x magnification. Plant phosphorus concentration was determined using the molybdenum blue method, while we determined chlorophyll concentration using the ethanol extraction spectrophotometry method (Arnon, 1949; Murphy and Riley, 1962).

Mature leaves, particularly the third leaf from the top, were selected for cell ultrastructure analysis. The leaves were washed with deionized water and blotted dry with absorbent paper. Using a scalpel, tissue samples of uniform size (1 mm thick, 1 mm long, and 2 mm wide) were quickly excised from the same location on each leaf. The samples were immediately placed in a 2.5% glutaraldehyde solution precooled to 4°C and fixed for 12 h. After fixation, the glutaraldehyde solution was discarded. The samples were rinsed with phosphate-buffered saline and then post-fixed in 1% osmium tetroxide solution. After dehydration and embedding, ultrathin sections were prepared and stained. The ultrastructure of the plant samples was observed using a transmission electron microscope (TEM).

We determined leaf MDA concentration using the thiobarbituric acid (TBA) method (Heath and Packer, 1968) and POD activity based on the guaiacol reduction method (Senthilkumar et al., 2021). For the fresh leaf samples, SOD activity was measured using the Nitroblue Tetrazolium (NBT) reduction method (Ge et al., 2022), and CAT activity was tested using the hydrogen peroxide method (Aebi, 1984).

Plant V concentration was determined using inductively coupled plasma optical emission spectrometry (ICP-OES; iCAP 7200, Thermo Fisher Scientific (China) Co., Ltd., China).

### Statistical analysis

We assessed normality and homogeneity of variance for all data. Data were subjected to a two-way ANOVA with inoculation (+AMF and -AMF) and V stress (low, medium, and high level) as variables. The *post hoc* test was conducted with Tukey’s HSD test. The experimental data were analyzed using SPSS statistical software (SPSS Inc., Chicago, USA). Bar charts and scatter plots were created using Origin Pro 2024b (Origin Lab Corporation, USA).

## Results

### AMF colonization of plant roots

We did not obtain evidence of AMF colonization in roots of the -AMF control. The percentage of root length colonization of the +AMF treatment was over 29% (**Supplementary Figure S1**). Furthermore, we found that AMF colonization decreased with soil V level. Specifically, the percentages of root length colonization with +AMF were 46% (± 2%), 39% (± 0.8%), and 29% (± 0.7%) for low, medium, and high soil V levels, respectively.

### Leaf ultrastructure

**Figure 1:**
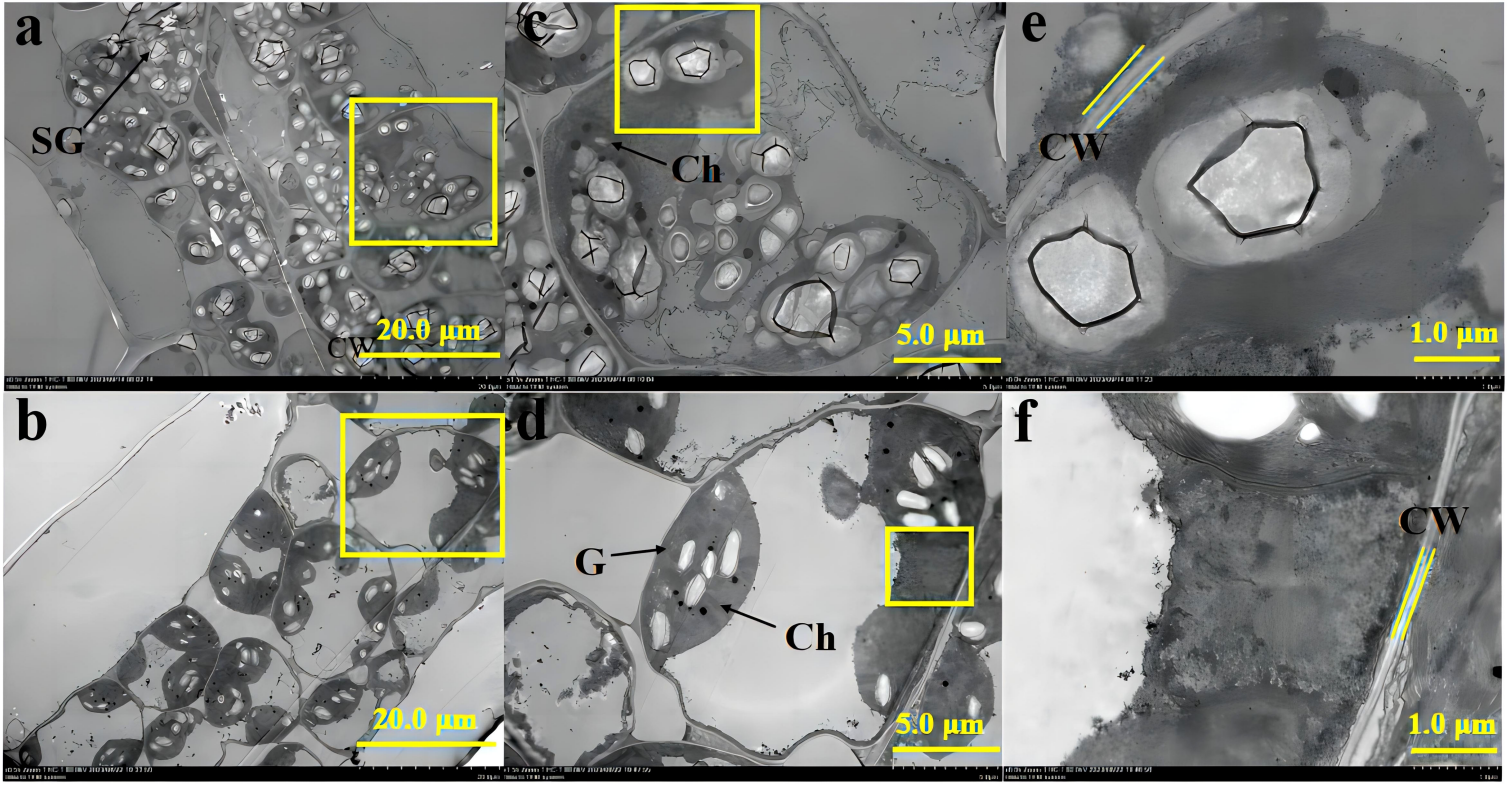
The transmission electron micrographs of leaf ultrastructure of -AMF **(a, c, e)** and +AMF **(b, d, f)** leaves under high soil V level. SG, starch granules; Ch, chloroplast; CW, cell wall; M, mitochondria; G, grana.

The transmission electron micrographs obtained with TEM represent the qualitative differences in leaf ultrastructure between +AMF and -AMF plants under high soil V level (**Figure 1**).

At high soil V levels, inoculation with AMF improved leaf cell ultrastructure. In particular, the ultrastructural images of -AMF leaf cells showed distortion of cell walls, poor development of cell membranes, and many more starch granules (**Figures 1a, c, e**). In contrast, +AMF leaf cells showed well-developed cell membranes, intact chloroplasts, and fewer but distinct starch granules (**Figures 1b, d, f**). No grana remained in the chloroplasts of -AMF and the onset of membrane disruption was observed; while those of +AMF showed a larger number of grana and the structure of grana and the chloroplasts was still preserved (**Figures 1c, d**). Compared to -AMF, +AMF mitochondria exhibited no signs of disruption, while the mitochondria in -AMF leaves were not visible (**Figures 1c, d**). The cell wall of −AMF leaf cells was thicker than that of +AMF leaf cells (**Figures 1e, f**).

**Figure 2:**
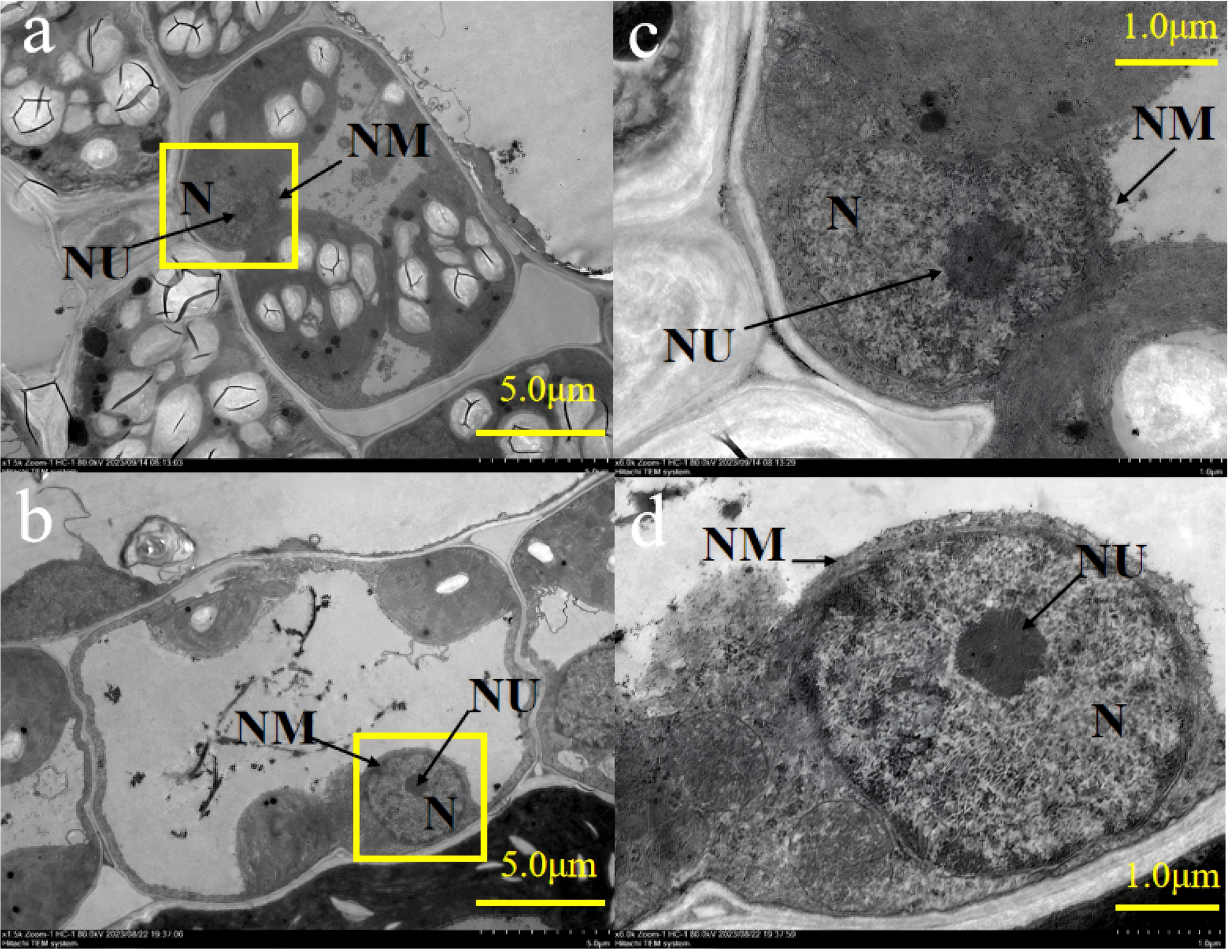
The transmission electron micrographs of nucleus in leaf cells of -AMF **(a, c)** and +AMF **(b, d)**, under high soil V level. N, nucleus; NU, nucleolus; NM, nuclear membrane.

**Figure 2** showed that the nucleus at +AMF had prominent nucleoli and chromatin material enveloped by an intact double-layered continuous nuclear membrane. In contrast, the nucleoli of -AMF were not clearly visible and the nuclear membrane of -AMF was not continuous.

### Leaf chlorophyll concentrations

**Figure 3:**
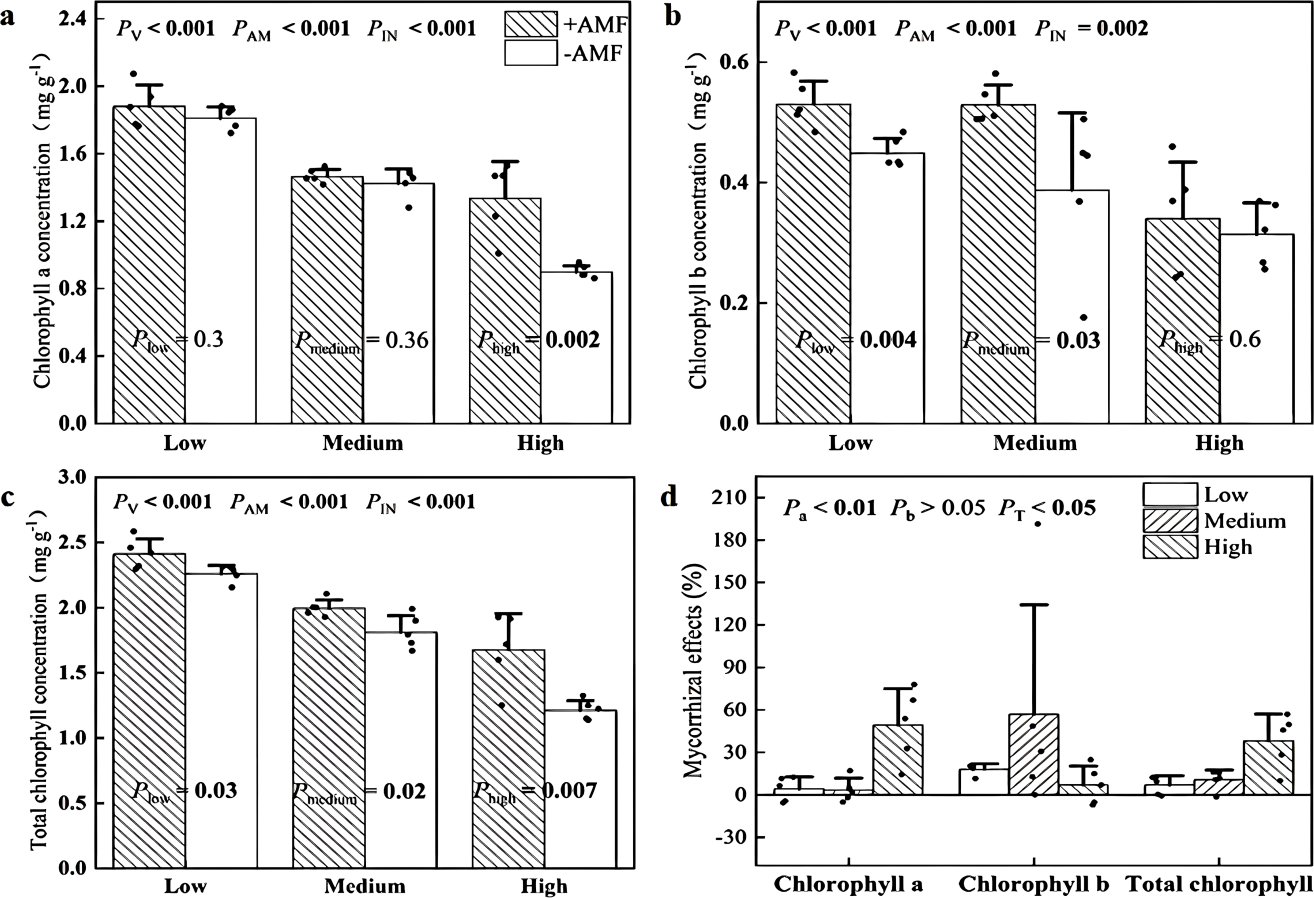
Leaf chlorophyll a concentration **(a)**, chlorophyll b concentrations **(b)**, total chlorophyll concentrations **(c)** of + AMF and -AMF plants and the mycorrhizal effects **(d)** under three soil V levels. *P*_V_, *P*_AM_, and *P*_IN_: significance of the differences among three V levels, between +AMF and -AMF treatments and the interaction between the two factors, respectively. *P*_a_, *P*_b_, and *P*_T:_ significance of differences in the mycorrhizal effects on chlorophyll a, chlorophyll b, and total chlorophyll concentrations under three soil V levels; the error bars were calculated as the standard deviations.

Inoculation with AMF and soil V levels significantly altered the concentrations of leaf chlorophyll a (**Figure 3a**), chlorophyll b (**Figure 3b**) and the total of chlorophyll a and b (**Figure 3c**). Importantly, significant interactions between these two factors were also tested (**Figures 3a–c**). Specifically, +AMF had 49% higher concentrations of chlorophyll a in leaves at high soil V levels ​​and 18% and 36% higher chlorophyll b concentrations at low and medium soil V levels, respectively. We calculated the mycorrhizal effects on chlorophyll a and b concentrations and found that the values of chlorophyll a were greatest at high soil V level.

### Plant oxidative damage and antioxidant system

**Figure 4:**
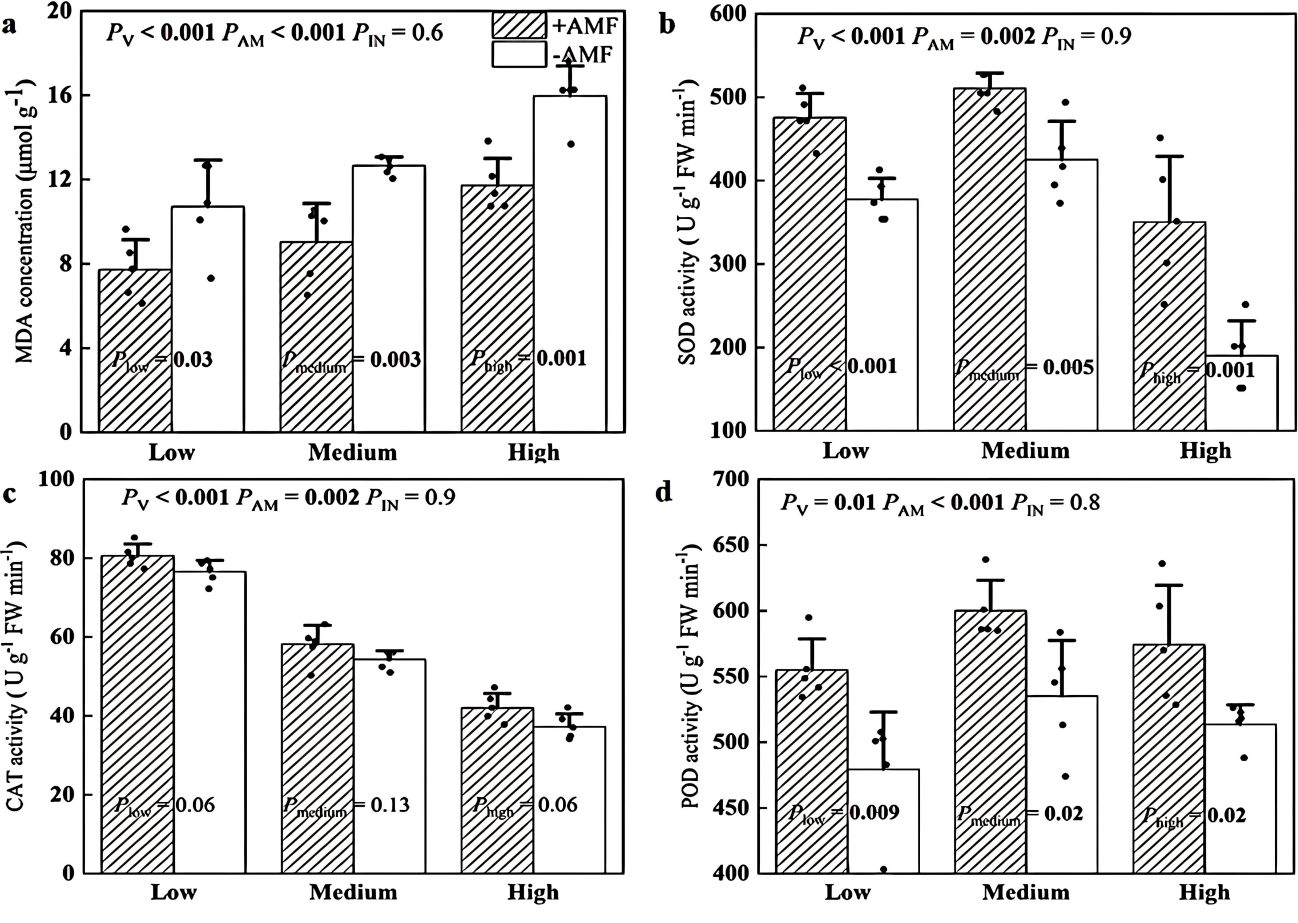
The MDA concentrations **(a)**, the SOD activity **(b)**, the CAT activity **(c)**, and the POD activity **(d)** of +AMF and -AMF leaves under different soil V levels. The set V concentrations of the low, medium, and high levels were 150 mg kg^-1^, 500 mg kg^-1^ and 1000 mg kg^-1^, respectively; -AMF and +AMF: non-inoculated and inoculated with AMF, respectively; *P*_V_, *P*_AM_, and *P*_IN_: significance of the differences among three V levels, between +AMF and -AMF treatments and the interaction between the two factors, respectively. *P*_low_, *P*_medium_, and *P*_high:_ significance of differences between -AMF and +AMF treatments under three V levels; the error bars were calculated as the standard deviations.

The MDA concentrations of +AMF leaves were 36%-40% lower than those of -AMF leaves under the three soil V levels (**Figure 4a**). We also found that +AMF plants exhibited 20%-84% higher activities of SOD (**Figure 4b**), 5%-13% greater activities of CAT and 12%-16% higher activities of POD (**Figure 4d**), regardless of the soil V level.

### Plant growth and phosphorus nutrition

**Figure 5:**
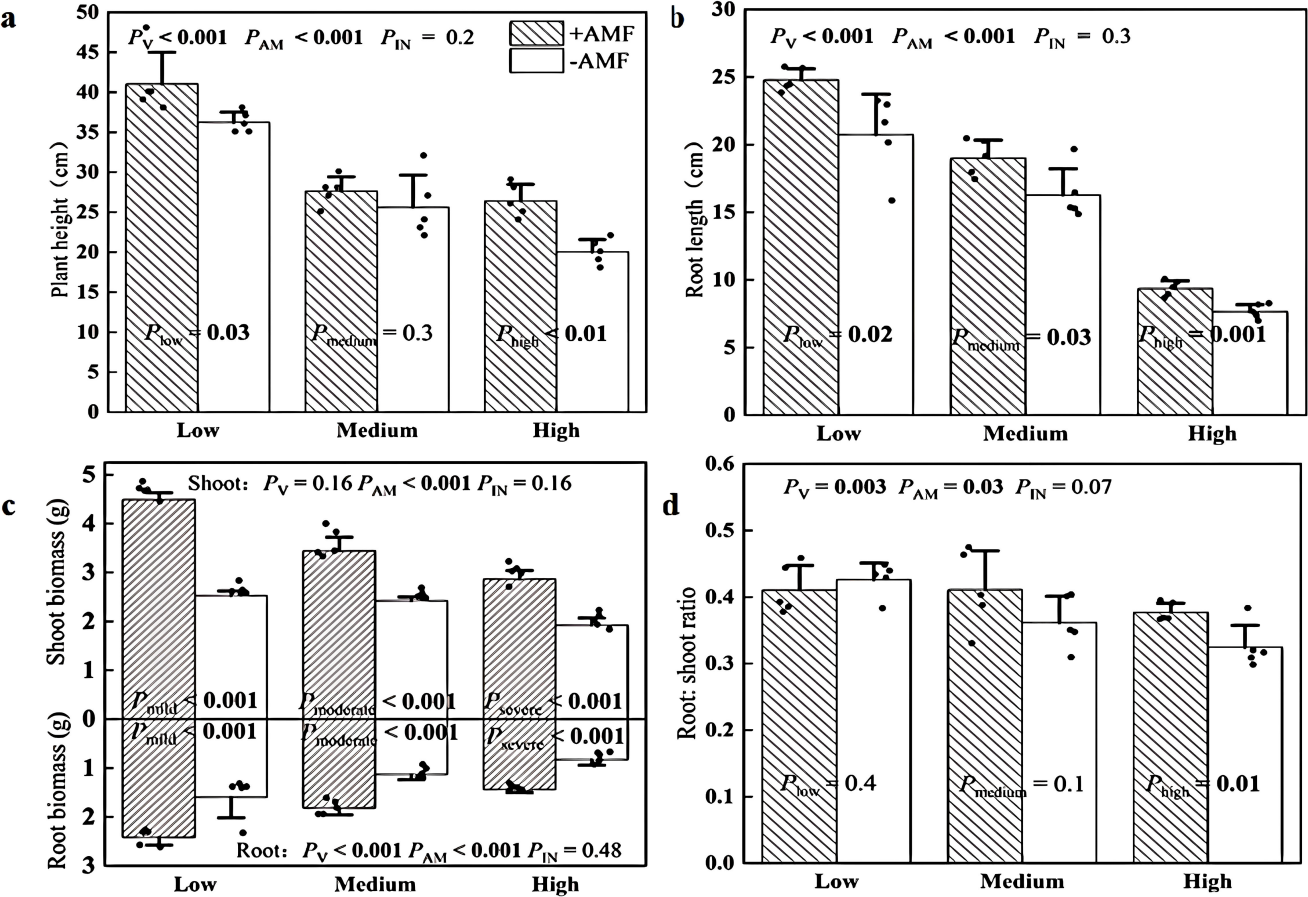
Plant height **(a)**, root length **(b)**, biomass **(c)** and root: shoot ratio **(d)** of + AMF and -AMF plants under three soil V levels. The set V concentrations of the low, medium, and high levels were 150 mg kg^-1^, 500 mg kg^-1^ and 1000 mg kg^-1^, respectively; -AMF and +AMF: non-inoculated and inoculated with AMF, respectively; *P*_V_, *P*_AM_, and *P*_IN_: significance of the differences among three soil V levels, between +AMF and -AMF treatments and the interaction between the two factors, respectively. *P*_low_, *P*_medium_, and *P*_high:_ significance of differences between -AMF and +AMF treatments under three soil V levels; the error bars were calculated as the standard deviations.

+AMF plants exhibited 13% and 32% greater plant height than -AMF plants under low and high soil V levels, respectively (**Figure 5a**; **Supplementary Figure S2**). There was no significant difference in plant height between +AMF and -AMF plants at medium soil V levels ​​(**Figure 5a**; **Supplementary Figure S2**). The roots of +AMF plants were 17%-23% longer than those of -AMF plants, regardless of soil V levels (**Figure 5b**; **Supplementary Figure S3**). +AMF plants had 42%-78% larger shoot biomass and 61%-73% greater root biomass than their -AMF counterparts across all three soil V levels (**Figure 5c**). Furthermore, +AMF plants had 16% larger root: shoot ratio at high soil V level (**Figure 5d**). The phosphorus concentrations of +AMF plants were 7%-13% higher than those of the -AMF control, regardless of soil V levels (**Supplementary Figure S4**).

### Plant V concentration and content

**Figure 6:**
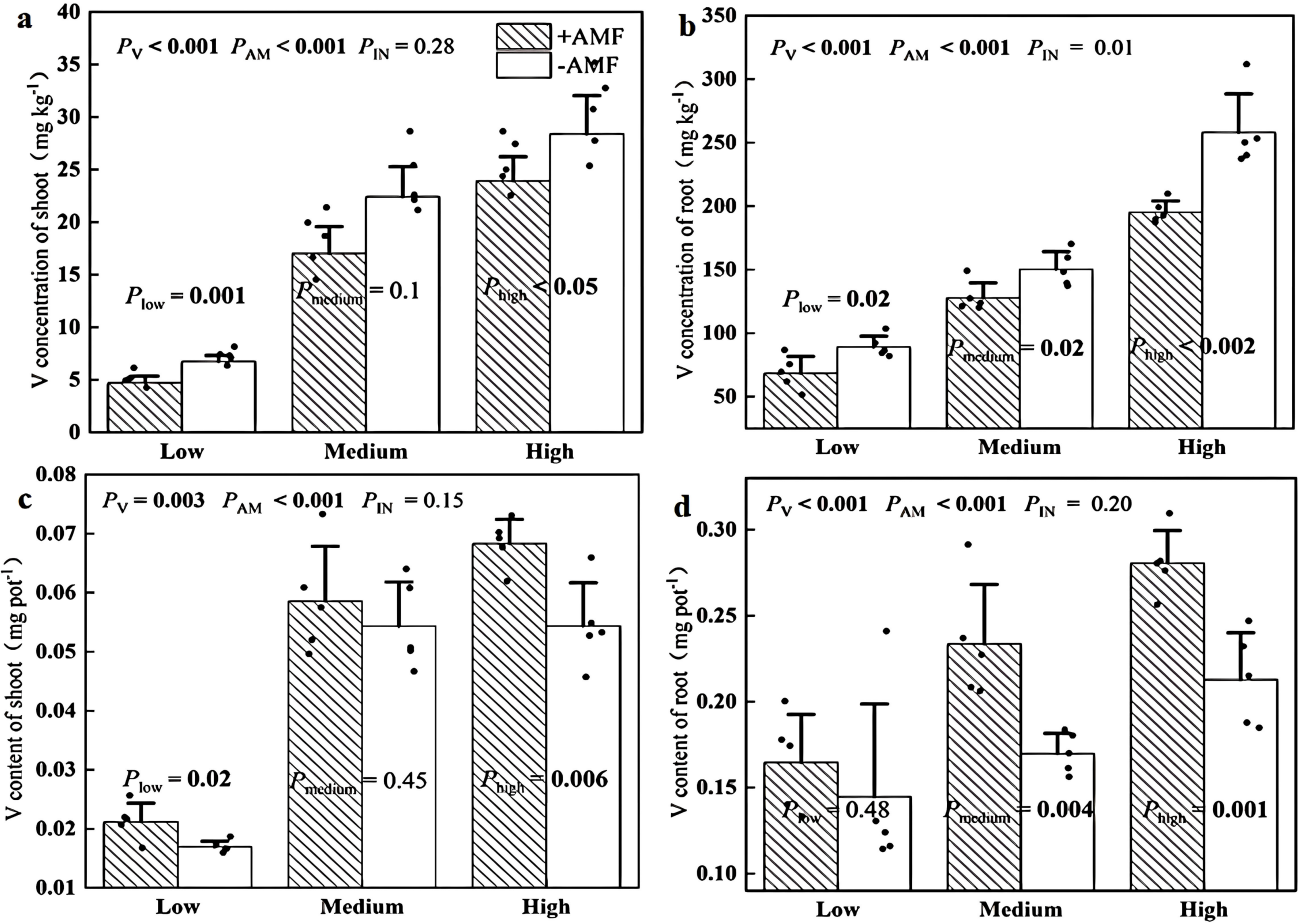
Effects of AMF on V concentration of shoot **(a)** and root **(b)** and on V content of shoot **(c)** and root **(d)**. The set V concentrations of the low, moderate, and high soil levels were 150 mg kg^-1^, 500 mg kg^-1^ and 1000 mg kg^-1^, respectively; -AMF and +AMF: non-inoculated and inoculated with AMF, respectively; *P*_V_, *P*_AM_, and *P*_IN_: significance of the differences among three V levels, between +AMF and -AMF treatments and the interaction between the two factors, respectively. *P*_low_, *P*_medium_, and *P*_high_: significance of differences between -AMF and +AMF treatments under three V levels; the error bars were calculated as the standard deviations.

**Figure 6** showed that +AMF shoots had 16%-30% lower V concentrations than -AMF plants while +AMF roots exhibited 52%-73% smaller V concentrations than the -AMF control. We also calculated the V content by multiplying the V concentration by the biomass. We found that the V content of +AMF shoots was 8%-26% higher than that of -AMF shoot while +AMF roots exhibited 14%-38% greater V content than their -AMF counterparts, although this difference existed for shoot V content at medium soil V level and the root V level was not significant at low soil V level.

## Discussion

Our results established *Rhizophagus irregularis* as a possible symbiont for V phytoremediation, enabling the green foxtail plant to thrive at 1000 mg kg^-1^ soil V (**Figure 5**). Contrary to the view of AMF as passive metal filters (González-Guerrero et al., 2008; Chen et al., 2018), our data revealed AMF’s active role in enhancing host tolerance to V stress with a hierarchical defense framework: physical defense (protection of leaf ultrastructure) → biochemical protection (prevention of chlorophyll downregulation and upregulation of antioxidant system) → system regulation (growth dilution effect). The consistent mycorrhizal effect across V concentrations (150–1000 mg kg^-1^) suggests that *Rhizophagus irregularis* targets fundamental detoxification framework rather than V load-specific adaptations. This is different with other studies where the effect of AMF on the tolerance of pigeon pea declines above 25 mg kg^-1^ under cadmium stress and 1000 mg kg^-1^ under molybdenum stress (Bisht and Garg, 2022; Zhang M. et al., 2023).

### AMF protected the ultrastructure of leaf cells

We found that *Rhizophagus irregularis* protected cell walls, cell membrane, chloroplasts, mitochondria, and cell nucleus of green foxtail under V stress (**Figures 1**, **2**). The protected leaf ultrastructure formed physical defense. This role of *Rhizophagus irregularis* was also found in other stains of AMF under cadmium stress. For example, *Rhizophagus intraradices* maintained the complete cell morphology of soybean leaves (Wang X. et al., 2023) and roots of *Potentilla sericea* (Feng et al., 2023) under cadmium stress; *Funneliformis mosseae* alleviated the symptoms of chloroplast damage and recovered the chloroplasts to normal size and shape in rice leaves under cadmium stress (Li et al., 2020). Evidence that protection of leaf cell ultrastructure by AMF to ensure optimal leaf cell function is needed in future studies. Despite this, we proposed that the role of AMF in protecting the ultrastructure of plant leaves and roots under heavy metal stress might not be species-specific for AMF or element-specific for heavy metals. This indicated a new idea for the application of multiple AMF strains in the remediation of soil with multi-metal composite pollution. AMF maintained the formation of chlorophyll

Photosynthetic pigments are fundamental to the process of photosynthesis and their concentration can reflect the growth status of plants (Zhu et al., 2024). In our study, as V stress increased, leaf concentrations of chlorophyll a and chlorophyll b decreased, leading to a decrease in total chlorophyll concentration (**Figure 3**). This negative effect of V stress on chlorophyll was also observed on tomato seedlings (Altaf et al., 2022). This may be because V inhibits the formation of precursors necessary for chlorophyll synthesis (Yuan et al., 2024). Notably, our data showed that *Rhizophagus irregularis* increased the concentrations of chlorophyll a, chlorophyll b and total chlorophyll, which led to a biochemical protection in mycorrhizal plants. This positive effect of *Rhizophagus irregularis* on chlorophyll concentrations of was also observed in wetland plant (*Iris wilsonii*) under chromium stress (Hu et al., 2020). Like the protection of plant ultrastructure, the role of *Rhizophagus irregularis* in enhancing chlorophyll was not species-specific as this role of *Funneliformis mosseae* and *Glomus mosseae* was also reported (Yang et al., 2022; Li et al., 2023). This positive effects of AMF on chlorophyll might be due to the induced expression of two enzymes involved in the chlorophyll biosynthetic pathway, i.e. coproporphyrinogen oxidase and Mg-protoporphyrin IX chelatase.

### AMF improved the antioxidant system

MDA is an oxidative product of plant membrane lipids, serves as a key indicator of lipid peroxidation and reflects the extent of oxidative damage in plant cells under V stress (Altaf et al., 2022). In our study, *Rhizophagus irregularis* significantly reduced leaf MDA concentrations by 27%-28% under all three V levels ​​(**Figure 4a**). Similar results were reported in a study (Zhang H. et al., 2023) in which cadmium stress was shown to increase MDA concentrations in green foxtail leaves, while *Funneliformis mosseae* decreased MDA concentrations. Another study showed that corn treated with *Funneliformis mosseae* and iron compounds under arsenic stress had a significant reduction in MDA levels, which is also consistent with our findings (Zhou et al., 2023). Therefore, these results provided evidence that AMF play a crucial role in protecting plant membrane lipids from oxidative damage under stress of heavy metals such as V, cadmium, and arsenic.

Our data indicated that *Rhizophagus irregularis* increased the activities of key antioxidant enzymes such as SOD (by 26%–43%), CAT (by 5%–13%), and POD (by 13%–16%) (although the differences in some cases were not significant). The activation of enzymatic antioxidant system in mycorrhizal plant also provided a biochemical protection from V stress. The effects of these AMF strains were different on the activities of key antioxidant enzymes under cadmium stress: *Rhizoglomus intraradices* gave the maximum boost to antioxidant defense system, especially SOD than those supplemented with *Funneliformis mosseae*, *Claroideoglomus claroideum*, and *Claroideoglomus etunicatum* (Bisht and Garg, 2022). Our results are consistent with previous studies conducted with other plans under V stress: *Funneliformis mosseae* increased the activities of SOD, POD and CAT in *Medicago sativa* leaves (Qiu et al., 2021); *Rhizophagus irregularis* played this role in C3 (rye) and C4 (sorghum) plants (Selim et al., 2021). Whether these AMF strains activate the non-enzymatic antioxidant system (including glutathione, ascorbic acid, carotenoids, and soluble proteins) under V stress need to be tested in the future studies.

### AMF have a growth dilution effect

We found that as soil V pollution increased, plant height, root length, and shoot and root biomass decreased (**Figures 5a–c**). This was consistent with another study that reported that alfalfa biomass was much lower at high soil V than at low soil V (Gan et al., 2020). Nevertheless, Chinese cabbage biomass increased with soil V concentrations (from 122 mg V kg^-1^ to 372 mg V kg^-1^), while plant growth was not measurable at high soil V concentrations (622 mg V kg^-1^) (Tian et al., 2014). A study also reported that at a soil V concentration of 900 mg kg^-1^, the alfalfa biomass was either immeasurable or the plants died (Wu et al., 2023). In our study, we found that the green foxtail was still able to grow even when the V concentration was set up to 1000 mg kg^-1^ (**Figure 5**). The above difference in effects of V may depend on soil V concentration and the plant species (Aihemaiti et al., 2017). Further research is in need to test whether there is an interaction between plant species and V stress.

Importantly, inoculation with *Rhizophagus irregularis* at the same V stress increased these indicators of plant growth, indicating improved tolerance to V stress in our study (**Figure 5**). This is consistent with a study that reported that inoculation with *Funneliformis mosseae* in citric acid-treated soils significantly improved shoot and root biomass of *Medicago sativa* (Qiu et al., 2021). Notably, we found the interaction between AMF inoculation and soil V level was not significant for these plant growth indicators (**Figure 5**). This meant that the mycorrhizal effect of *Rhizophagus irregularis* on plant growth was relatively stable at low, medium, and high V levels. The positive effect of this AMF strain on plant growth might be attributed to the improvement in P nutrition (**Supplementary Figure S4**). In particular, the mycorrhizal effect on total biomass was 70% -85%, higher than that of the V-resistant bacterial strain (22%-24%) and the V-resistant endophytic bacterium *Serratia marcescens* PRE01 (7%-10%) (Wang et al., 2018; Gan et al., 2020). These discrepancies in results could be due to other biotic factors such as microbial species and plant species, or to abiotic factors such as soil V level. Therefore, we call for studies that compare the effect of these microbes on plant growth under V stress under the same circumstances.

Our results showed that plants inoculated with *Rhizophagus irregularis* had lower V concentrations as well as higher biomass and V content in both shoots and roots (**Figure 6**). This is consistent with previous findings indicating that this AMF strain increased V concentrations, plant biomass and V content in shoots and roots of rye and sorghum (Selim et al., 2021). The lower metal concentrations, greater biomass, and higher metal content observed in plants inoculated with AMF compared to those uninoculated formed a growth dilution effect (Chen et al., 2004). The concentration-independent mycorrhizal effect positions *Rhizophagus irregularis* as a robust bioinoculant for heterogeneous V-polluted sites, contrasting with Cd systems requiring strain-dose optimization (Liu et al., 2015).

## Conclusions

This study elucidates the hierarchical defense framework by which *Rhizophagus irregularis* enhances *Setaria viridis* tolerance to V stress, offering novel insights into AMF-mediated heavy metal resistance. AMF preserved cell membrane, chloroplast, and mitochondria of plant leaves under V stress, a critical adaptation for maintaining photosynthetic efficiency. Its biochemical protection included prevention of chlorophyll downregulation (increasing leaf total chlorophyll concentration by 7%-38%) and upregulation of antioxidant system (reducing oxidative damage by 36%-40%). Biomass-driven V dilution contributed 42-78% to total tolerance while plant V accumulation increased by 8-38%. The AMF-mediated tolerance remained consistent across V concentrations (150-1000 mg kg⁻¹), challenging the classical dose-effect paradigm. These findings redefine AMF functionality in heavy metal remediation, emphasizing their role in stabilizing redox-active pollutants. The hierarchical framework provides a blueprint for designing AMF-assisted phytoremediation of V-polluted soils. Our study did not explore the failure threshold of AMF at extremely high concentrations of V (>1000 mg kg^-1^), nor did it evaluate the interference of polymetallic complex pollution (such as V-Cd coexistence) on the symbiotic system. In addition, the field trial data still need to be supplemented to verify the laboratory conclusion.

## Data Availability

The raw data supporting the conclusions of this article will be made available by the authors, without undue reservation.
