# Supplementary material for: The concentration-independent effect of arbuscular mycorrhizal fungi on the tolerance of green foxtail to vanadium stress
Source: Front Plant Sci. 2025 May 27;16:1592931. doi: 10.3389/fpls.2025.1592931 (PMC12150089; doi:10.3389/fpls.2025.1592931)
Supplement: Supplementary file 1 [file DataSheet1.docx]

Supplementary Material

**The concentration-independent effect of arbuscular mycorrhizal fungi on the tolerance of green foxtail to vanadium stress**

Shujuan Zhang^a^, Yuexiao Dong^a,b^, Jingfan Qi^a,b^, Jinlong Wang ^a,b^, Ze Xi^a^, Ziwei Cao^a^, Kinjal J. Shah^a^, Zhaoyang You^a,b*^

Shujuan Zhang: Zhangshujuan525@sina.com (Associate Professor)

Yuexiao Dong：dongyuexiao1@163.com

Jingfan Qi: qijingfan98@163.com

Jinlong Wang: 459783987@qq.com

Ze Xi ：202461224024@njtech.edu.cn

Ziwei Cao: czw200138@163.com

Kinjal J. Shah: [kjshah@njtech.edu.cn](mailto:kjshah@njtech.edu.cn)

Zhaoyang You (corresponding author): youzhaoyang@njtech.edu.cn (Professor)

^*^ Corresponding author

Email: youzhaoyang@njtech.edu.cn

Postal address: Room 405, Shangde Building, College of Urban Construction, Nanjing Tech University, Puzhu Road(S) 30, 211816, Nanjing, China

^a^ College of Urban Construction, Nanjing Tech University, Puzhu Road(S) 30, 211816, Nanjing, China;

^b^ Nanjing Yuqing Environmental Technology Co., Ltd, Nanjing 211500, China.

Catalogue

[Fig. S1 Images of mycorrhizal colonization of green foxtail (*Setaria viridis*)*.* (× 200 microscope) 3](#_Toc191309395)

[Fig. S2 Plant growth of the -AMF (a) and +AMF group (b) under high V pollution 3](#_Toc191309396)

[Fig. S3 Root length of the +AMF and -AMF groups 4](#_Toc191309397)

[Fig. S4 Phosphorus concentrations of plant leaves under different V stresses 4](#_Toc191309398)


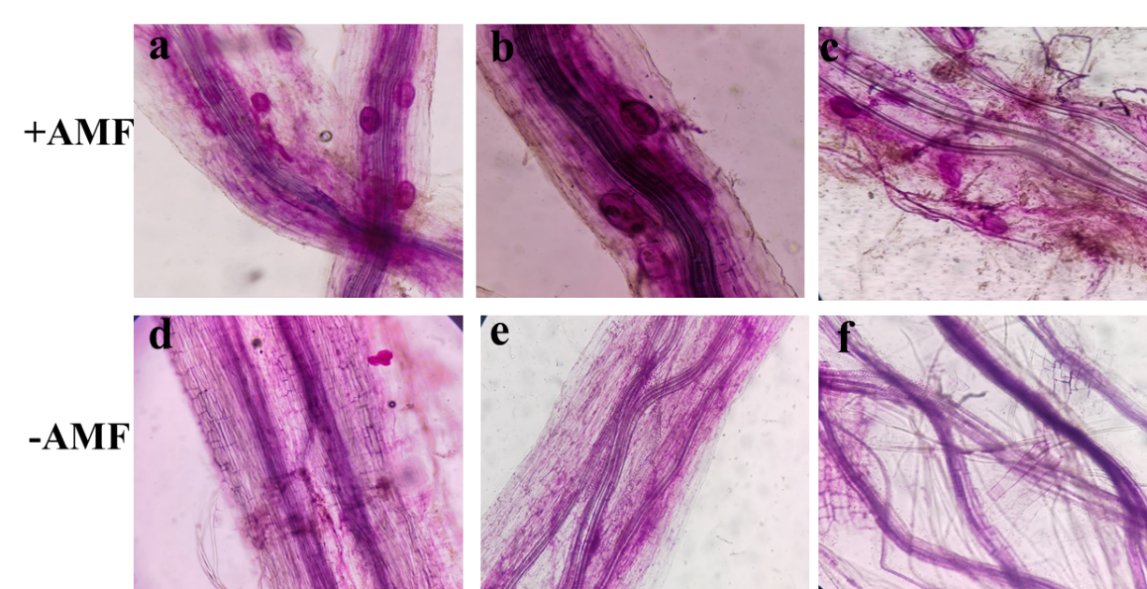


Fig. S1 Images of mycorrhizal colonization of green foxtail (*Setaria viridis*)*.* (× 200 microscope)

Note: a, d are lowly V-contaminated soils, b, e are mediumly vanadium-contaminated soils, c, f are highly vanadium soil treatments, +AMF means arbuscular mycorrhizal inoculation treatment, -AMF means no inoculation treatment. (The same below)


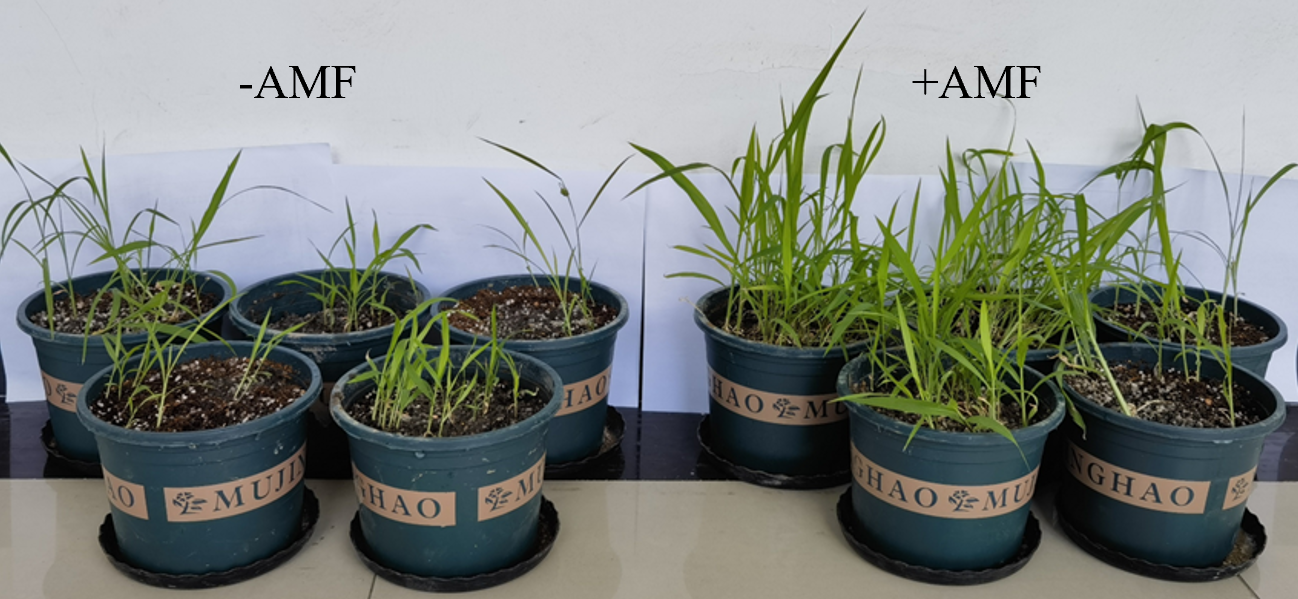


Fig. S2 Plant growth of the -AMF (a) and +AMF group (b) under high V pollution


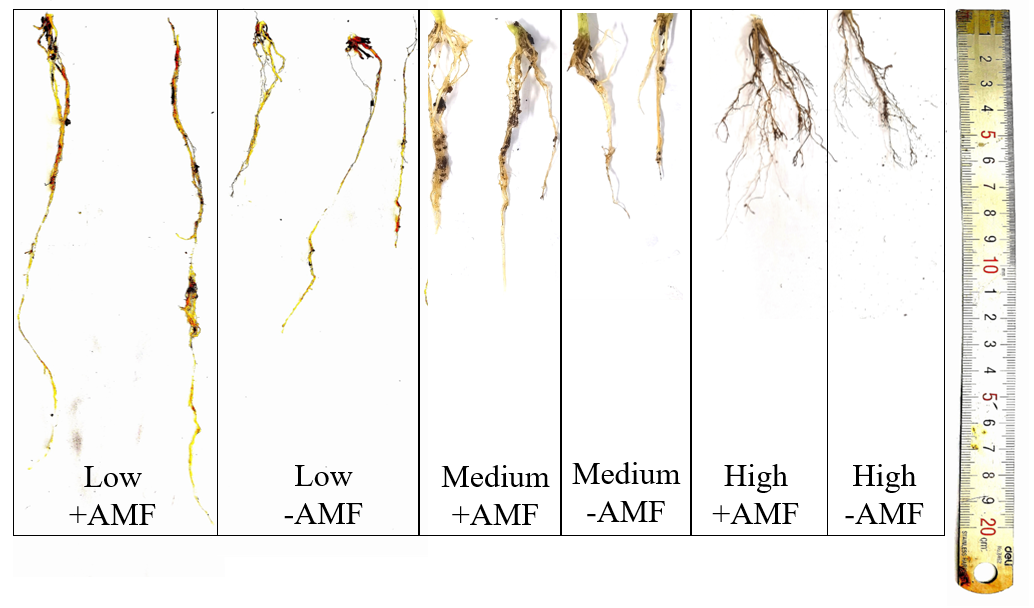


Fig. S3 Root length of the +AMF and -AMF groups


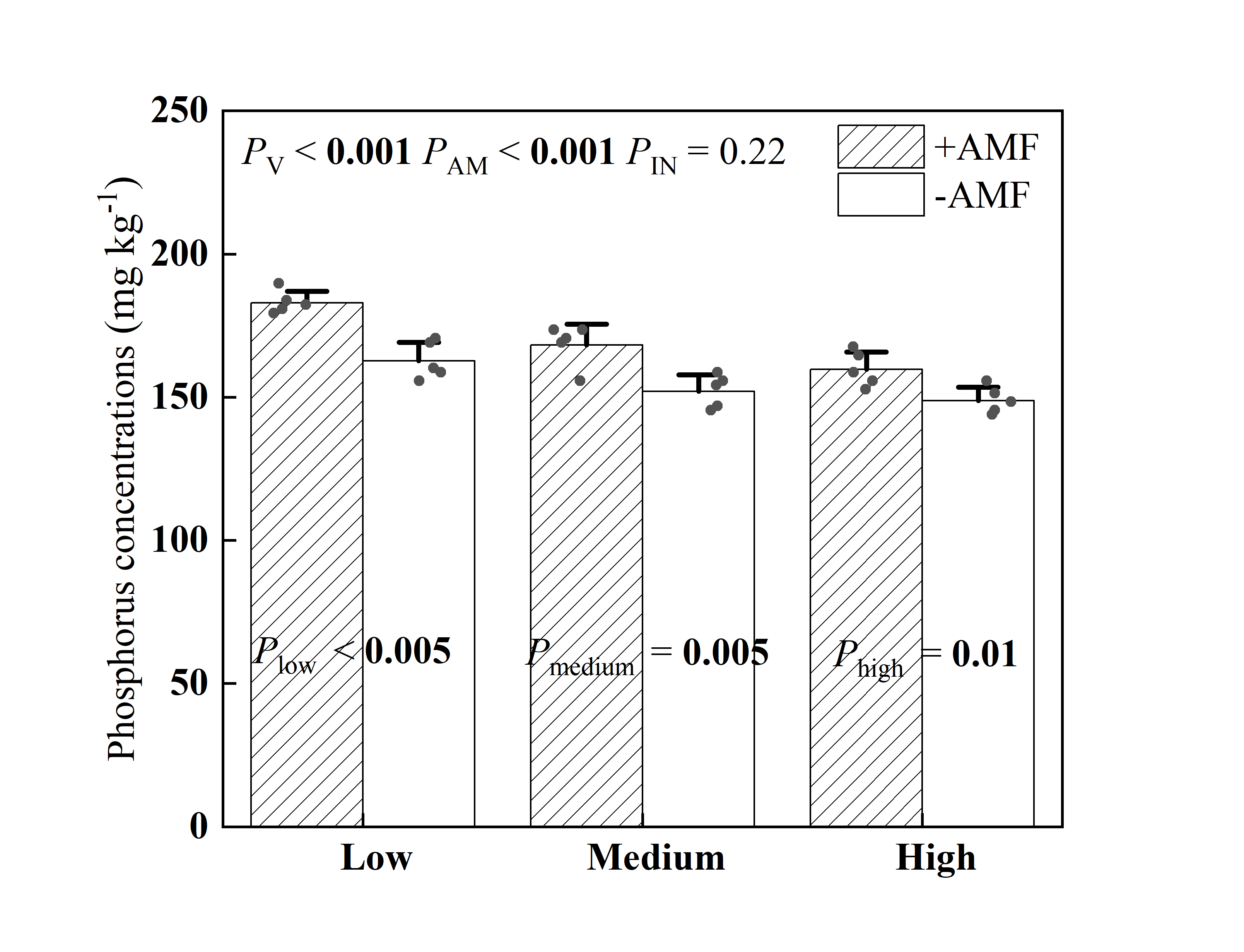


Fig. S4 Phosphorus concentrations of plant leaves under different V stresses

Note: *P*_V_, *P*_AM_, and *P*_IN_: significance of the differences among three V levels, between +AMF and -AMF treatments and the interaction between the two factors, respectively; *P*_low_, *P*_medium_, and *P*_high_

_:_ significance of differences between -AMF and +AMF treatments under three V levels; the error bars were calculated as the standard deviations.
